# Extraction of basic movement from whole-body movement, based on gait variability

**DOI:** 10.1002/phy2.49

**Published:** 2013-08-22

**Authors:** Christian Maurer, Vinzenz von Tscharner, Michael Samsom, Jennifer Baltich, Benno M Nigg

**Affiliations:** Human Performance Laboratory, Faculty of Kinesiology, University of CalgaryCalgary, Alberta, Canada

**Keywords:** Control strategies, kinematic, motor control, movement variability, preferred movement, step-to-step variability

## Abstract

The aim of this study was to quantify the step-to-step variability (SSV) in speed-variant and speed-invariant movement components of the whole-body gait pattern during running. These separate aspects of variability can be used to gain insight into the neuromuscular control strategies that are engaged during running. Ten healthy, physically active, male recreational athletes performed five treadmill running trials at five different speeds (range: 1.3–4.9 m/sec). The whole-body movement was separated into principal movements (PM) using a principal component analysis. The PMs were split into two groups: a speed-variant group, where the range of motion (amplitude of PMs) changed with running speed; and a speed-invariant group, where the range of motion was constant across various speeds. The step-to-step variability (SSV) of the two groups was then quantified. The absolute SSV was the summed variability across all gait cycles, whereas the relative SSV was the summed variability divided by the magnitude of the movement. The absolute SSV of the speed-variant movements increased with running speed. By contrast, the relative SSV of the speed-variant group (as normalized to the PM amplitude) decreased asymptotically toward a minimal level as running speed increased. Both the absolute and relative SSV of the speed-invariant movements revealed a minimum at 3.1 m/sec. The whole-body gait pattern during running can be subdivided into speed-variant and speed-invariant movements. An interpretation of the SSV based on minimal intervention theory suggests that speed-variant movements are more tightly controlled, as evidenced by a lower degree of variability compared to the speed-invariant movements.

## Introduction

Bernstein suggested that the fluctuations in cyclic movements like walking, running, and cycling are not because of sloppiness of the system, but because of a well-defined feedback system (Bernstein 1967). The observation from Bernstein has been supported by optimal control models, which suggest that fluctuations in task-relevant movement components are reduced, whereas fluctuations in task-irrelevant components are uncontrolled and, therefore, larger (Todorov 2004).

In running fluctuations can be characterized by quantifying the step-to-step variability (SSV). Because the instrumental noise level of state-of-the-art motion-capture systems is only a fraction of the amplitude of a recorded movement (Richards 1999), it is commonly believed that the SSV in cyclical movements reflects fluctuations caused by the neuromuscular system (Winter 1984; Schoner and Kelso 1988; Hamill et al. 1999; Keith et al. 2001; Jordan et al. 2006; Braun et al. 2010; Manor et al. 2010; Wolpert et al. 2011).

Gait variability has been studied with respect to both movement efficiency – defined as the minimum energy cost per unit distance – and movement pathologies (Brisswalter and Mottet 1996; Hamill et al. 1999; Keith et al. 2001; Ivanenko et al. 2007; Seay et al. 2011), as illustrated by the following examples. With regard to movement efficiency, one study found that during walking, the SSV related to stride frequency is smallest at one's preferred walking speed (Jordan et al. 2006). Another study compared the walking patterns of young healthy adults to those of old healthy adults and young adults with neurological disorders. Both long- and short-term correlations showed that the walking patterns of young healthy adults exhibited a higher degree of local dynamic stability (Dingwell and Marin 2006). In other studies, an increase in SSV related to stride frequency was associated with an increase in falls in elderly populations (Maki 1997; Hausdorff et al. 2001). With regard to movement pathologies, individuals suffering from conditions such as patellofemoral pain syndrome were found to exhibit less SSV than those without that condition (Hamill et al. 1999; Keith et al. 2001; Heiderscheit et al. 2002). In people suffering from lower back pain, the pelvis was found to move more in phase with the trunk, and this resulted in reduced SSV (Seay et al. 2011). Such studies indicate that movement variability is more than simple “noise” to be eliminated. Rather, it may be evidence of a functional property of the human motor system. Indeed, given the importance of movement variability from this perspective, it has been suggested that movement training with kinematic assessment might be more beneficial for patients with spinal cord injuries than training of individual muscles (Ivanenko et al. 2009; Lacquaniti et al. 2012).

To date, gait variability research has focused primarily on discrete variables such as stride length or stride frequency (Borghese et al. 1996). However, because human movement is characterized by the interaction of several limbs, and because the nervous system controls whole-body movement, it is important to consider whole-body movement when investigating aspects of motor control. Within the context of movement preparation, the activation of several muscles has previously been investigated (Krishnamoorthy et al. 2003; Bizzi et al. 2008; Valero-Cuevas et al. 2009; Enders et al. 2013). As several muscles cross the same joints, a given movement can be achieved through multiple combinations of muscle activation. Analyzing whole-body movement, therefore, may lead to a more complete understanding of the final output of motor control. Mathematical approaches such as principal component analysis (PCA) have made it possible to comprehensively evaluate whole-body movement during walking and running, as well as allowing the possibility to extract and analyze individual movement features in a high-dimensional movement space (Mah et al. 1994; Troje 2002; Daffertshofer et al. 2004; Vallery and Buss 2006; Federolf et al. 2012; Maurer et al. 2012).

Separating a complex movement into specific movement components makes it possible to analyze and compare those components to see if they reflect the same movement characteristics. Movement components are defined here as movement patterns where the movements of different limbs correlate with each other in time. As variability has been associated with aspects of motor control, movement variability can be used to characterize the motor control output of different movement components. If two movement components show the same variability characteristics, then it is possible that they are controlled in a similar way. If there are significant differences, however, it may be that the controls for the two movement components have different origins. One can see that the combined analysis of whole-body movement with the analysis of movement variability can be used to gain a better understanding of movement in general, and can indicate differences in the underlying control mechanism(s) of human locomotion. For instance, the amount of variability in components of repeated movements can be used to label these movement components as task relevant and task irrelevant (Kutch and Valero-Cuevas 2012). Based on the minimal intervention theory, the task relevant movement components are tightly controlled and, therefore, show lower variability in the movement (Tresch and Jarc 2009; Valero-Cuevas et al. 2009; Dingwell et al. 2010). It should be noted that variability can provide an indication of the similarity in the underlying control mechanisms but not which controller is used for the movement itself.

In view of these possibilities, we explored the feasibility of combining a whole-body approach with movement variability analysis to analyze running at different velocities. It has previously been shown that center-of-mass movement is speed invariant (Lee and Farley 1998), whereas the sagittal movement of the limbs is speed variant. Therefore, we separated the whole-body movement of running into speed-variant and speed-invariant movement components, and characterized the control of those components using a new method to quantify movement variability.

The purposes of this study were to (Bernstein 1967) use PCA on a full-body marker set in order to define a vector space of running movements, (Bizzi et al. 2008) determine if the trajectories of the running movement projected onto different base vectors differ in the speed dependency, and (Borghese et al. 1996) characterize the variability in the speed-variant and speed-invariant movement components. We hypothesized (H1) that: whole-body movement can be separated into speed-variant and speed-invariant principal movements, and (H2) that the characteristic SSV in speed-variant movements is smaller than the variability in the speed-invariant movement because the speed-variant movements change with running speed and are, therefore, expected to be the relevant movement components. In accordance with the minimal intervention theory, we expected a smaller SSV in these movements.

## Methods

### Subjects

Ten healthy, physically active, male recreational athletes (age 25.4 ± 5.4 years, mass 75.8 ± 11.3 kg, height 180.8 ± 5.1 cm, 10 km running speed 3.08 ± 0.43 m/sec, mean and standard deviation [SD]) participated in this study. Athletes were included if they ran at least twice a week for more than 30 min per running session and were rear foot strikers. The 10 km running speed was determined from their most recent competition time. Athletes ran in their own shoes. None of the athletes who participated in this study used minimalist footwear. All subjects provided written informed consent in accordance with the University of Calgary's policy on research using human subjects. The study protocol was approved by the Conjoint Health Research Ethics Board at the University of Calgary.

### Experimental protocol

All subjects performed treadmill running at five different speeds (1.3 m/sec, 2.2 m/sec, 3.1 m/sec, 4.0 m/sec, and 4.9 m/sec), wearing their own running shoes and apparel. This broad range of running velocities was included in order to better understand the effects running speed. Subjects were instructed to perform at all speeds with a running style characterized by the presence of a flight phase. The subjects warmed up by running on the treadmill at a self-selected speed for 10 min. After warming up, each subject started at the lowest speed (1.3 m/sec). The speed was increased in 0.9 m/sec increments up to 4.9 m/sec. Subjects ran for at least 40 sec at each speed, with data recorded during the last 30 sec of each run. Kinematic data were collected at 240 Hz from 47 reflective markers, using an eight-camera motion-capture system (EVaRT, Motion Analysis Corporation, Santa Rosa, CA). The markers were placed on both sides of the body (five on each foot, three on each shank, three on each thigh, and four on the pelvis), as well as one on the sternum, two on the spine (T1 and L3), and four on the head. In addition, the locations of the ankle, knee, hip, shoulder, elbow, and wrist joints were indicated on the lateral side of each limb with one marker each (Fig. 2).

### Analysis

**Figure 1:**
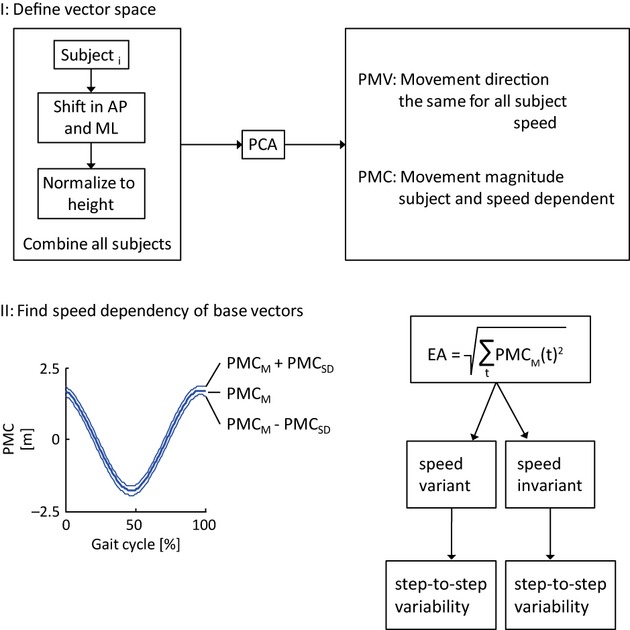
Flow chart of the analysis. Kinematic data from each subject were preprocessed before they were combined in one matrix. A PCA was applied onto this matrix resulting in PMV and PMC. Based on the PMC, the effective amplitude EA was calculated. The relationship between the EA and speed was tested for every PM. Two groups were separated, a speed-variant and a speed-invariant one. The step-to-step variability in each group was calculated from the SD of the PMC. For details of the calculations see text.

**Figure 2:**
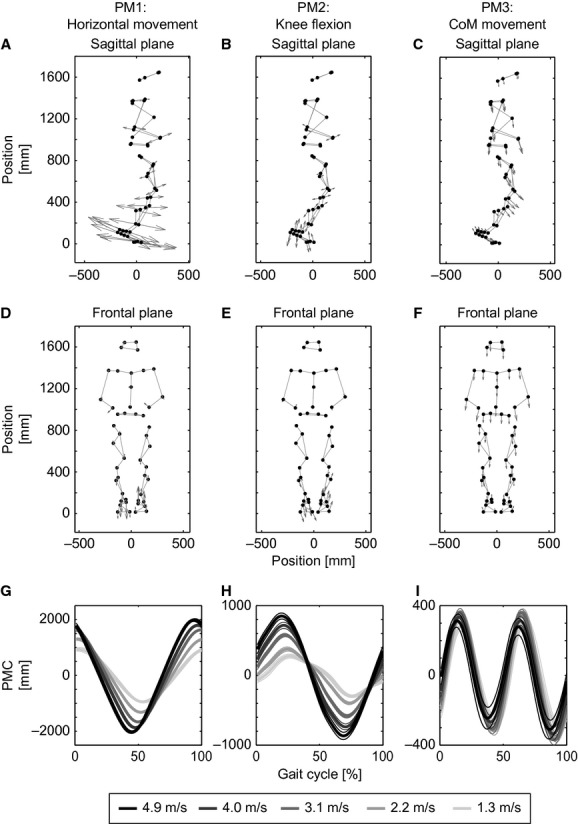
(A–C) First three PMs and range for one subject at 3.1 m/sec in the sagittal plane. (D–F) First three PMs and range for one subject at 3.1 m/sec in the frontal plane. PM1 primarily describes the forward/backward movement of the limbs, whereas PM2 describes knee flexion and lifting of the feet. PM3 describes the center-of-mass (CoM) movement. The lengths of the arrows indicate the range of motion, *R*. The length was magnified by 2 for PM1 (A, D), 6 for PM2 (B, E), and 20 for PM3 (C, F). Note that the direction of the PMV is the same for all subjects and speeds, however, the range, *R*, is subject and speed variant. (G–I) PMC waveforms for one subject for the five different speeds (from light to dark: 1.3, 2.2, 3.1, 4.0, 4.9 m/sec) and PMs 1, 2, and 3. Mean and SD of the PMC waveforms are plotted. The middle line of the waveforms is the mean; the outer lines are mean ± SD.

A general flow of the mathematical data processing can be seen in Figure 1. The details of PCA are explained elsewhere (Federolf et al. 2012; Maurer et al. 2012). In summary, however, the kinematic data for this study consisted of the 3D positions of the markers in space (47 markers × three dimensions = 141 marker positions). All marker positions were represented as vectors, with the length of the vector equal to the number of time points. The marker positions were sampled at 240 Hz for a period of 30 sec, yielding a vector length of 7200 data points. The markers were shifted in the AP and ML direction to the center of the pelvis. The position vectors were normalized to the height of the subjects (Fig. 1). The vectors that were measured for all five speeds and all 10 subjects span a vector space called *pattern space*, and were stored in a matrix (M) that was used in the PCA (Fig. 1). The marker positions for any vector in pattern space can be displayed in the sagittal (Fig. 2, A–C) and frontal (Fig. 2, D–F) planes, showing the movement pattern. The kinematic data were separated into a mean pattern representing the average position of the marker during the gait cycle (black points in Fig. 2, A–F), and correlated deviations from the mean pattern computed by the PCA applied to M (Fig. 2, A–F, direction of the arrows). The correlated deviations from the mean pattern were called *principal movements* (PMs). PMs were represented by vectors called *principal movement vectors* (PMVs), and are the orthonormal eigenvectors obtained during the PCA, sorted according to the eigenvalues. The eigenvalues indicate how much variability in the original data is explained by each PMV. The PMVs form a base of the pattern space with a dimensionality that is limited to the rank of the covariance matrix of M.

The PMVs do not, however, represent time evolution. The time evolution of the movement was represented by *principal movement components* (PMCs), which were calculated by projecting the kinematic data stored in M onto the PMVs. These projections represent the time-variant contribution to the variability (waveforms) and, thus, to the movement (Fig. 2, G–I). The PMC are the combined movement of all markers in the direction of a specific PMV. The specific movement of the PMVs can be characterized within vector plots (Fig. 2, A–F). Individual waveforms for gait cycles (identified by the lowest vertical position of the heel marker) were extracted from the PMCs and were time normalized by a resampling algorithm. All normalized waveforms had a length of 100 points. When the normalized waveforms are multiplied by the corresponding PMVs and added to the mean pattern, the result shows part of the whole-body movement restricted to one PM. The same can be done for groups of PMVs, but this can only be displayed in a dynamic picture. The magnitude of the movement was indicated by representing the movement for each PMV as a vector arrow originating at each marker. The length of the arrow indicates the relative amplitude of the marker within one movement component (Fig. 2, A–F).

When performing a PCA, one usually considers only the first few PMVs and disregards the later ones, which explain only a fraction (smaller than 5%) of the total variance. Information about significant differences, however, has often been found in the lower ordered PMs (Maurer et al. 2012); therefore, significant information about the movement is also found in these lower ordered PMs. For this study, all calculated PMs were used, and groups of PMs that reflected similar speed dependencies were combined.

### Computation of the step-to-step variability

**Figure 3:**
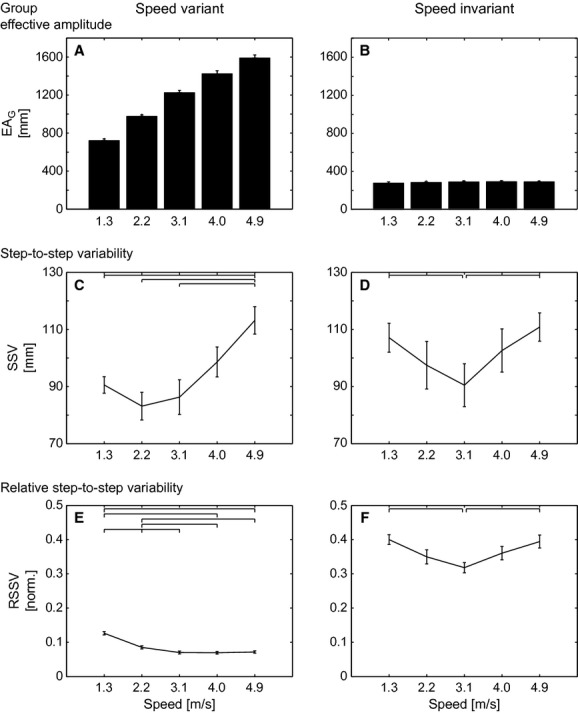
Group-specific properties. (A, C, E) PMs where the square of the range, R_G_^2^, increased with running speed (speed-variant movements). (B, D, F) PMs where the square of the range, R_G_^2^, did not show a correlation with speed (speed-invariant movements). (A, B) Mean and SE of the range, R_G_, summed over the PMs for one group. (C, D) Mean and SE of the SSV. (E, F) Mean and SE of the RSSV. Horizontal bars indicate significant difference in the mean value of the variables between the speeds. *Significant tests (ANOVA):* SSV speed variant (C): F(4,45) = 6.1 (*P* < 0.001); SSV speed invariant (D): F(4,45) = 2.59 (*P* = 0.049). RSSV speed variant (E): F(4,45) = 32 (*P* < 0.001); RSSV speed invariant (F): F(4,45) = 2.66 (*P* = 0.045).

Based on the time-normalized PMC, means of waveforms (PMC_M_) and standard deviations (PMC_SD_) were computed at each time point. These values (PMC_M_ and PMC_SD_) can be displayed as a time series (Fig. 2, G–I). The effective amplitude (EA) of the PMC waveforms was computed as the root mean square (RMS) across the PMC_M_ waveforms. Thus, there was one EA value per subject, speed, and PM. The PMs were separated into two groups: one for EA^2^ values that showed a positive linear correlation with speed (the speed-variant group), and one for EA^2^ values that showed no linear correlation with speed (the speed-invariant group). The EA^2^ value was used as it represents the variance of each PM and as variances are additive. The EA^2^ values from each group were summed across the PMs, resulting in the square of the group effective amplitude (EA_G_^2^) for each group, subject, and speed. The mean of EA_G_ and its standard error (SE) were computed across all subjects (Fig. 3, A–B). The square of the group effective amplitude, EA_G_^2^, is the variance of the movement within one group, and was used to calculate the contribution of the group to the whole movement.

The SSV is a measure for the variability in the movement between different gait cycles. The SSV was calculated individual for each subject and is therefore a measure for how much the whole-body movement of each subject fluctuates during running. The SSV was the RMS of all SDs of the PMC waveforms across 100 time points per subject, speed, and PM. The SSV^2^ was summed across the PMs belonging to one group, resulting in the square of the step-to-step variability per group (SSV_G_^2^). There was one SSV_G_^2^ value per subject, speed, and group. The mean and SE of the SSV_G_ across all subjects were calculated for each speed and group (Fig. 3, C–D). The relative step-to-step variability (RSSV_G_) was the SSV_G_ normalized by the EA_G_ value. One RSSV_G_ value was calculated for each subject, speed, and group. The mean and SE of the RSSV_G_ values were calculated across all subjects (Fig. 3, E–F).

### Statistics

Pearson linear correlation analysis was used to analyze the speed-variant changes in EA^2^. The SSV_G_ and RSSV_G_ were tested for normal distribution with a Lilliefors test. A one-way analysis of variance (ANOVA) was used to analyze differences in the SSV_G_ and RSSV_G_ with respect to speed. A Tukey–Kramer post hoc test was used to identify the speeds where the mean values of the variables showed a significant difference. For all tests, a significance level of α = 0.05 was used.

## Results

The rank of the matrix was 139 indicating that there were 139 PMs in order to describe the whole pattern space. The first PM (PM1) is mainly characterized by forward/backward-directed PMVs (Fig. 2A). The movement vector has large horizontal components in the sagittal plane (Fig. 2A), and small vertical components in the frontal plane (Fig. 2B). This movement represents the horizontal movement of the legs and arms. The second PM (PM2) is characterized by the vertical movement of the feet. This can best be seen in the frontal plane (Fig. 2E). The third PM (PM3) is the vertical movement of the trunk and can be identified as the center-of-mass (CoM) movement (Fig. 2, C and F). PM1 and PM2 are examples of speed-variant movements. The maximal amplitude of the PMCs for these PMs increased with increasing speed, as did the EA (Fig. 2, G and H). The maximal amplitude of the PMCs of PM3 did not change with speed (Fig. 2I). Higher ranked PMs characterize more subtle features.

The correlation analyses revealed that 31 PMs showed a correlation between the EA^2^ and speed, whereas 108 PMs did not show a correlation between EA^2^ and speed (Table 1). EA^2^ increased for the speed-variant group and was constant for the speed-invariant group. In the speed-variant group, the total movement captured by the PMVs increased with speed, whereas the amount of the movement explained by the speed-invariant group decreased as speed increased (Table 2). The combination of the speed-variant PMs is characterized by a horizontal movement (Video S1, combination of the speed-variant PMs). The combination of the speed-invariant PMs is characterized by a vertical movement (Video S2, combination of the speed-invariant PMs).

**Table 1:** Group-specific separation of PMs

Group	Distribution of PMs across the 139 dimensions of the pattern space
Speed-variant PMs	1, 2, 4, 5, 10, 12, 16–21, 23, 25, 26, 28, 30, 37, 39, 57, 59–62, 65, 67, 69, 77, 83, 90, 97;
Speed-invariant PMs	3, 6–9, 11, 13–15, 22, 24, 27, 29, 31–36, 38, 40–56, 58, 63, 64, 66, 68, 70–76, 78–82, 84–89, 91–96, 98–139;

In the speed-variant group, the square of the range, *R*^2^, of a PM showed a linear correlation with speed. In the speed-invariant group, no correlation was found between the *R*^2^ value of a PM and speed. It is significant that both groups are represented across the full pattern space, showing that speed affected amplitude for all running trials.

**Table 2:** The amount of movement variance explained by the two groups

Speed (m/sec)	1.3	2.2	3.1	4.0	4.9
Speed variant	87.5%	92.5%	95.0%	96.2%	97%
Speed invariant	12.5%	7.5%	5.0%	3.8%	3.0%

The mean across all subjects of the movement EA_G_ for the speed-variant group increased from 0.72 m at 1.3m/sec to 1.6 m at 4.9 m/sec. The average value of the movement EA_G_ for the speed-invariant group over all speeds and subjects was 0.279 m (Fig. 3, A and B).

The SSV_G_ for the speed-variant group was lowest at 2.2 m/sec. Significant differences were found between the speeds 1.3 m/sec and 4.9 m/sec, 2.2 m/sec and 4.9 m/sec, and 3.1 m/sec and 4.9 m/sec (Fig. 3C). No significant differences were found between the four lower speed levels. The minimum SSV_G_ for the speed-invariant group was at 3.1 m/sec. This minimum was significantly different from the lowest speed (1.3 m/sec) and the highest speed (4.9 m/sec) (Fig. 3D). When both groups were combined, the minimum of the combined variability remained at 3.1 m/sec.

The RSSV_G_ decreased for the speed-variant group. RSSV_G_ values for the first three speeds were significantly different. The RSSV_G_ values at 1.3 m/sec and 2.2 m/sec were significantly different to those at 4.0 m/sec and 4.9 m/sec. The RSSV_G_ for the speed-invariant group was lowest at 3.1 m/sec. The RSSV_G_ values at 1.3 m/sec and 4.9 m/sec were significantly different to those at a speed of 3.1 m/sec.

## Discussion

The results show that a whole-body running movement can be decomposed by a PCA into PMs. When visually represented, these PMs illustrate specific movement characteristics such as arm and leg movements in the sagittal plane, vertical CoM movement, and foot-specific movements (Fig. 2A–F). It was possible to assign all PMs to speed-variant and speed-invariant groups of variables, confirming our first hypothesis, H1, that “whole-body movement can be separated into speed-variant and speed-invariant principal movements.” This result confirms previous findings that the CoM movement is speed invariant, whereas the stride length is speed variant (Lee and Farley 1998). Of the variables analyzed, 108 were determined to be speed-invariant, and 31 were determined to be speed-variant principal movements.

The variability in the two movement components showed different speed dependencies. The SSV_G_ for the speed-variant movement component increased as running speed increased, whereas the relative step-to-step variability (RSSV_G_) for the same group decreased across the lower running speeds and reached a plateau across the higher running speeds (Fig. 3, C and E). The speed-invariant movement component, however, showed a minimum for both the SSV_G_ and the RSSV_G_ (Fig. 3, D and F). Thus, the results confirm our second hypothesis, H2, that “the characteristic SSV of speed-variant and speed-invariant movements is different.” The relative SSV reflects the variability in the movement per unit length, and can therefore be compared between the two movement components.

The minimum in the SSV_G_ and the RSSV_G_ appears at the speed of the 10k running speed of the subjects. It could be speculated that this minimum exists because the athletes train most of the time around this speed. Therefore, the feedback gain in the control sequence is optimized for this running speed and the movement pattern exhibit therefore the smallest fluctuations in these range. As the running speed is the running speed where the subjects trained most, it might be speculated that this is also the speed where the movement is performed with the highest efficiency in terms of motor control. The minimum of the RSSV_G_ of the speed-invariant movements might therefore be related to the highest efficiency of the motor control system.

The results showed that the relative SSV for the speed-variant movement component was approximately four times smaller than the relative SSV in the speed-invariant movement component. The differing magnitude in relative SSV can be interpreted in two ways. First, within the framework of minimal intervention theory (Todorov 2004), our findings suggest that speed-variant principal movements are more tightly controlled by the human motor system than speed-invariant principal movements. Furthermore, minimal intervention theory posits that task-relevant movement components are more tightly controlled than nontask-specific movements. Thus, it might be argued that for running, speed-variant movement components are task-relevant, whereas speed-invariant movement components are not.

Extrapolating from this to application in running training, then, emphasis should be placed on coordinating speed-variant movements (movements in the sagittal plane). Center-of-mass movements, on the other hand, appear to be less significant in terms of movement control.

The second interpretation for the differing magnitude in relative SSV focuses on movement constraints. It has been shown that the organization of a muscle-tendon unit limits or defines the number of possible movements available for accomplishing a specific task (Kutch and Valero-Cuevas 2012). Relative SSV may be understood as an indicator of the number of movement solutions available to perform a task. The number of solutions for a particular movement task depends on the biomechanical constraints of the limbs. Less RSSV might, therefore, reflect a less complex movement and a lower requirement for or corresponding reduction in neurological control effort. For the data presented here, then, it might be argued that because the RSSV was shown to decrease as running speed increased, the control effort for the movement also decreased. However, a certain RSSV remained even at the highest speeds – necessary, perhaps, to be able to compensate for sudden disturbances. Within the context of this interpretation, the decrease in RSSV seen at higher running speeds reflects a lower number of movement solutions and a less complex movement. This interpretation is consistent with current views in the literature (Winter and Eng 1995).

It may be possible to differentiate between neuronal control aspects and biomechanical constraints through nonlinear analysis of the time signal. Analysis such as sample entropy (Newell 1993; Keith et al. 2001) provides information about the short- and long-term structure of the time signal. Nonlinear analysis was not performed as part of this study, however, because our data set was only 30 sec long. It has been shown that for a representative analysis of long- and short-term correlations, at least 100 steps (and therefore up to 2 min of data) are required (Herman et al. 2005; Lamoth et al. 2009; Terrier and Dériaz 2011). In future studies, this limitation should be addressed by recording longer time sequences and by applying nonlinear methods to different components of whole-body movement.

In summary, this study showed that it is possible to take whole-body principal movements and extract those that reflect the speed-variant and speed-invariant EAs of the running movement, respectively. We conclude that the two identified (speed-variant and speed-invariant) movement components are controlled by at least two different mechanisms. We have provided two different interpretations for our findings, one based on the internal control mechanisms of the human body and one based on the biomechanical constraints of the movement. Differentiation between these two interpretations may be possible with nonlinear analysis of the time series. This study provides the foundation for analyzing different aspects of human movement based on whole-body analysis. This analysis intrinsically incorporates coupling between different limbs and has, therefore, the potential to show that the control mechanisms for specific movement components could be different. We conclude that speed-variant and speed-invariant aspects of movements should be investigated separately, as the control characteristic measured by the variability is different for these two movement components. In principal, this method can be applied to other cyclic movements, opening new possibilities for investigating the characteristic of variability and its relationship to the output of human motor control.

## Abbreviations

CoM: Center of mass
EA: Effective amplitude
EA_G_: The group effective amplitude
EA_G_^2^: The square of the group effective amplitude
M: Matrix; used in the PCA
PCA: Principal component analysis; a mathematical approach use in this study to evaluating whole-body movement and separating or extracting individual movement features in a high-dimensional movement space
PM: Principal movement, organized (for this study) into two groups: speed variant and speed invariant; a correlated deviation from the mean pattern computed by the PCA
PMC: Principal movement component; the representation of the evolution in time of a movement
PMC_M_: Mean of waveform, based on a time-normalized PMC
PMC_SD_: Standard deviation of waveform, based on a time-normalized PMC
PMV: Principal movement vector; a vector that represents a principal movement in pattern space; the orthonormal eigenvector obtained during the PCA, sorted according to the eigenvalues
R: Range of motion
R^2^: The square of the range of motion
R_G_: The range of motion per group
R_G_^2^: The square of the group range of motion
RMS: Root mean square
RSSV: Relative step-to-step variability
RSSV_G_: Relative step-to-step variability, determined from the SSV_G_ normalized by the EA_G_ value
SD: Standard deviation
SE: Standard error
SSV: Step-to-step variability
SSV_G_: The step-to-step variability per group
SSV_G_^2^: The square of the step-to-step variability per group

## Supporting Information

**Video S1.** Video that demonstrates the speed-variant component of running. mp4

**Video S2.** Video that demonstrates the speed-invariant component of running. mp4
